# Formylpeptide receptor 2: Nomenclature, structure, signalling and translational perspectives: IUPHAR review 35

**DOI:** 10.1111/bph.15919

**Published:** 2022-07-29

**Authors:** Cheng Xue Qin, Lucy V. Norling, Elizabeth A. Vecchio, Eoin P. Brennan, Lauren T. May, Denise Wootten, Catherine Godson, Mauro Perretti, Rebecca H. Ritchie

**Affiliations:** ^1^ Drug Discovery Biology, Monash Institute of Pharmaceutical Sciences Monash University Melbourne Victoria Australia; ^2^ William Harvey Research Institute, Barts and the London School of Medicine Queen Mary University of London London UK; ^3^ Diabetes Complications Research Centre, Conway Institute and School of Medicine University College Dublin Dublin Ireland

**Keywords:** ALXR, annexin‐A1, FPR, lipoxin A_4_, *N*‐formylated peptides, resolution of inflammation

## Abstract

We discuss the fascinating pharmacology of formylpeptide receptor 2 (FPR2; often referred to as FPR2/ALX since it binds lipoxin A_4_). Initially identified as a low‐affinity ‘relative’ of FPR1, FPR2 presents complex and diverse biology. For instance, it is activated by several classes of agonists (from peptides to proteins and lipid mediators) and displays diverse expression patterns on myeloid cells as well as epithelial cells and endothelial cells, to name a few. Over the last decade, the pharmacology of FPR2 has progressed from being considered a weak chemotactic receptor to a master‐regulator of the resolution of inflammation, the second phase of the acute inflammatory response. We propose that exploitation of the biology of FPR2 offers innovative ways to rectify chronic inflammatory states and represents a viable avenue to develop novel therapies. Recent elucidation of FPR2 structure will facilitate development of the anti‐inflammatory and pro‐resolving drugs of next decade.

**Abbreviations**
ALX: aspirin‐triggered LXA_4_ receptor
AMPK: AMP‐activated protein kinase
ANXA1: annexin‐A1
ChemR23: chemerin receptor 1/ Chemerin_1_
ECL: extracellular loop
fMLF: *N*‐formyl‐Met‐Leu‐Phe
RvD: resolvin
TM: transmembrane
WKYMVm: synthetic peptide Trp‐Lys‐Tyr‐Met‐Val‐D‐Met‐NH_2_

## 1 INTRODUCTION AND RECEPTOR NOMENCLATURE

Formylpeptide receptor 2 (FPR2/ALX) belongs to the formylpeptide receptor (FPR) subfamily of GPCRs. This family of chemoattractant pattern recognition receptors is expressed in mammalian phagocytic leukocytes and other cells of diverse lineage. FPRs are involved in host defence against pathogens via recognition of evolutionary conserved pathogen‐associated molecular patterns (PAMPs), in particular, *N*‐formylated peptides. In addition, FPRs sense damage‐associated molecular patterns (DAMPs) to respond to host‐mediated signals of cellular distress and dysfunction during non‐infectious inflammation (Migeotte et al., 2006; Roh & Sohn, 2018).

There are three human FPR‐subtypes, FPR1, FPR2 and FPR3, all encoded within a genomic cluster on chromosome 19q13.3 (Alexander, Christopoulos, et al., 2021; Bäck et al., 2021; Ye et al., 2009). FPR1 (formerly known as FPR) was the first subtype to be identified, as the high‐affinity target on neutrophils that recognises and mediates actions of N‐formyl‐Met‐Leu‐Phe (fMLF, often incorrectly referred to via the non‐standard abbreviation, fMLP) and its derivatives (Williams et al., 1977). FPR1 was first cloned in 1990 from a cDNA library constructed from dibutyryl‐cAMP‐differentiated human myeloid HL‐60 cells, revealing a 350‐amino‐acid residue polypeptide chain with two variants (Boulay et al., 1990). Shortly after, FPR2 (formerly known as the FPR‐like 1, FPRL1 or FPRH1) and FPR3 (formerly known as the FPR‐like 2, FPRL2 or FPRH2) were identified based on conserved amino‐acid sequence homology (69% for FPR2 and 56% for the FPR3) to human FPR1 (Bao et al., 1992; Ye et al., 1992). Within other mammals, differential selective pressures and gene expansion have resulted in varied members of the FPR family. The mouse genome encodes seven different subtypes (denoted as *Fpr*) with both overlapping and distinct functions to their human counterparts (Ye et al., 2009). Based on sequence homology, *Fpr1* is considered the equivalent of *FPR1* (77% similarity), while two mouse genes, *Fpr2* and *Fpr3*, are partial orthologues to human *FPR2* as they share high sequence identity and respond to lipoxin A_4_ (LXA_4_ ) (Vaughn et al., 2002; Ye et al., 2009). Fpr2 also binds F2L, a potent agonist for the human FPR3 (Gao et al., 2007). Mouse FPR3 displays similar intracellular distribution as observed with the human FPR3 (He et al., 2013), suggesting evolutionary correlation and possible convergence of function of the mouse FPR2/FPR3 to the human FPR2/FPR3. Four other mouse genes (*Fpr‐rs3*, *Fpr‐rs4*, *Fpr‐rs6* and *Fpr‐rs7*) encode chemosensory receptors (Rivière et al., 2009).

FPR2 differs from FPR1 in the greater diversity of the ligands it recognises, ranging from small molecules to peptides, proteins and lipids (He & Ye, 2017). Bacterial‐derived *N*‐formylpeptides, for which the subfamily of GPCRs is named, are potent agonists at FPR1 but display much lower affinity and efficacy at FPR2, despite a high degree of amino‐acid sequence overlap between the two receptors (Williams et al., 1977; Ye et al., 1992). Further pharmacological characterisation has identified higher affinity endogenous ligands for FPR2 including eicosanoid lipoxin A_4_ (LXA_4_) (Fiore et al., 1994), pro‐resolving lipid mediator resolvin D1 (Krishnamoorthy et al., 2010) and aspirin‐triggered 15‐epi‐lipoxin A_4_ (ATL) (Takano et al., 1997). This preferential binding of LXA_4_ relative to *N*‐formylpeptides per se led to alternative naming for FPR2, as the LXA_4_ and aspirin‐triggered LXA_4_ receptor (ALX or ALXR), such that FPR2/ALX has often been the nomenclature used (Alexander et al., 2021; Bäck et al., 2021; Ye et al., 2009). However, controversy has surrounded FPR2/ALX, due to its complex receptor biology. Incongruities in the literature have included conflicting data, depending on the animal model used, cell background (e.g. whether wildtype or transfected) and both source and solvent of the exogenously applied compound (e.g. high concentrations of DMSO). This has created debate around the ability of endogenous ligands (e.g. LXA_4_) to generate FPR2 signalling (de Gaetano et al., 2019, 2021; Merlin et al., 2022). It is possible there is a disconnect between heterologous‐ versus endogenous‐expressed FPR2 or allosteric modes of receptor interaction (Ge, Liao, et al., 2020), that once fully explored in light of publication of FPR2 structure (Chen et al., 2020; Zhuang et al., 2020), may provide an explanation for these apparent discrepancies. At present, in the style of NC‐IUPHAR where single receptor nomenclature is preferred, based on confirmed phylogeny and activation by *N‐*formylpeptides (albeit at higher concentrations), we refer to this receptor as FPR2 throughout the review.

## 2 BIOLOGY OF FPR2

**FIGURE 1:**
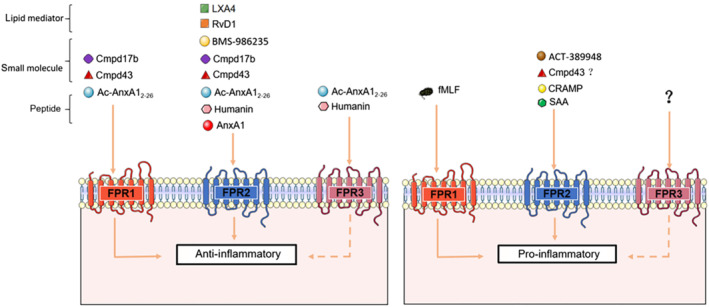
Summary illustration of the chemical diversity of FPR agonists across the three human receptor subtypes with their known anti‐inflammatory versus pro‐inflammatory consequences (see text for references). Abbreviations: Ac‐AnxA1_2‐26_, annexin 1‐(2‐26); Cmpd, compound; CRAMP, cathelicidin‐related antimicrobial peptide; RvD, resolvin; SSA, serum amyloid A.

FPR2 agonists include LXA_4_, the acute phase protein serum amyloid A (SAA), the amyloid beta 42 (Aβ_42_) peptide, the glucocorticoid‐modulated protein the glucocorticoid‐modulated protein annexin‐A1 (ANXA1), the acetylated N‐terminal peptide fragment of ANXA1, annexin 1‐(2‐26) [Ac‐AnxA1_2‐26_ ],the mitochondrial‐derived peptide humanin, resolvins such as RvD1, the synthetic hexapeptide synthetic peptide Trp‐Lys‐Tyr‐Met‐Val‐D‐Met‐NH_2_ (WKYMVm) and small molecules such as compound 43 and compound 17b (Table 1). FPR2 KO mice exhibit the impaired macrophage chemotaxis, abnormal neutrophil physiology, increased susceptibility to inflammatory disease (such as arthritis) and bacterial infection (Perretti & Godson, 2020). Further adding to the intriguing biology of this receptor family is their ability to be activated by both pro‐ and anti‐inflammatory agonists (Table 1, Figure 1). For example, both pro‐inflammatory fMLF and anti‐inflammatory annexin 1‐(2‐26) are FPR1 agonists, with opposing actions (Qin et al., 2015; Ye et al., 2009). The same applies to FPR2, where pro‐inflammatory serum amyloid A and cathelicidin‐related antimicrobial peptide (CRAMP, LL‐37 as human ortholog), as well as anti‐inflammatory annexin 1‐(2‐26) and LXA_4_, are all FPR2 agonists (Perretti et al., 2015; Qin et al., 2015). This promiscuity at FPRs, which confounds our understanding of this intriguing receptor family, is a recurring feature of this review (see Section 4). Both pro‐inflammatory and pro‐resolving agonists are essential for mounting the host response to pathogens and signalling their efficient removal, and thus both are critical for survival. Evidence that aspirin‐triggered 15‐epi‐lipoxin A_4_ I (ATL) is a biased allosteric modulator, also mediating inverse agonism at low concentrations (Ge, Zhang, et al., 2020), suggests a dual regulatory mechanism to exert its anti‐inflammatory effects. This, together with observations that LXA_4_ binds other GPCRs such as GPR32 (G protein‐coupled receptor 32; Krishnamoorthy et al., 2010), further adds to the signalling complexity of this lipid mediator. Likewise, it is noteworthy that while the endogenous agonist ANXA1 has been regarded as a non‐selective FPR1/FPR2 agonist (as is clear for its N‐terminal peptide annexin 1‐(2‐26)), evidence that human FPR1 actually binds full‐length ANXA1 protein at biologically relevant (sub‐micromolar) concentrations is lacking. For example, detectable binding of ANXA1 is only evident at human FPR1 at 10 μM (and not at lower concentrations), compared with its EC_50_ of 0.15 μM at human FPR2 (Hayhoe et al., 2006). For comparison, annexin 1‐(2‐26) exhibits an EC_50_ at both FPRs at ~1 μM (Hayhoe et al., 2006). The frequent interchange of the term ‘ANXA1’ with ‘annexin 1‐(2‐26)’ (e.g. in article titles), when the shorter peptide was actually studied (Walther et al., 2000), likely account for this anomaly, which is easy to address. Indeed, the depth of the binding pocket for FPR1 may not be conducive to larger peptides and proteins such as ANXA1, as opposed to the shorter tail of annexin 1‐(2‐26) (see Section 5).

**TABLE 1:** Current and former human FPR family receptor nomenclature and their agonists

Nomenclature		Known agonists	Molecule type	References
Systematic	Aliases and former names			
FPR1	FPR	Anti‐inflammatory: annexin 1‐(2‐26) Cmpd17b, Cmpd43	Peptide Small molecule	(Hayhoe et al., 2006) (Garcia et al., 2019; Qin et al., 2013, 2017)
		Pro‐inflammatory: fMLF	Peptide	(Showell et al., 1976)
FPR2	ALX, ALXR, ALX/FPR2, FPR2/ALX, FPRL1, FPRH1	Anti‐inflammatory: AnxA1 Humanin annexin 1‐(2‐26) LL‐37 LXA_4_, RvD1, RvD3 ATL, AT‐01‐KG Cmpd17b, Cmpd43 BMS‐986235	Protein Protein Peptide Peptide Lipid Small molecule Small molecule	(Perretti et al., 2002) (Ying et al., 2004) (Galvao et al., 2021) (Wan et al., 2011) (Arnardottir et al., 2016; Fiore et al., 1994; Krishnamoorthy et al., 2010) (Garcia et al., 2019, 2021; Qin et al., 2013, 2017) (Lee et al., 2009)
		Pro‐inflammatory: SAA CRAMP (LL‐37), amyloid beta 42 (Aβ_42_)	Protein Peptide	(He et al., 2003; Sodin‐Semrl et al., 2004) (Wan et al., 2011; Zhang, Gong, et al., 2020; Kurosaka et al., 2005).
FPR3	FPRL2, FPRH2	Anti‐inflammatory: Humanin annexin 1‐(2‐26)	Protein Peptide	(Ernst et al., 2004; Harada et al., 2004; Ying et al., 2004)
		Pro‐inflammatory: None known	Not applicable	(Harada et al., 2004; Ying et al., 2004)

Abbreviations: ATL; aspirin‐triggered 15‐epi‐lipoxin A_4;_ CRAMP, cathelicidin‐related antimicrobial peptide; Cmpd17b, compound 17b; Cmpd43, compound 43; RvD, resolvin; SAA, serum amyloid A.

FPR2 is well‐known for playing a role not only in mediating host defence against bacteria (Zhang, Gao, et al., 2020), but also regulating inflammation and its effective resolution by modulating inflammatory mediators, as well as cell‐surface receptors and enzymes, limiting leukocyte recruitment and stimulating phagocytic uptake, ensuring restoration of tissue function. The resolution of inflammation reflects several coordinated actions: ‐ reduced and/or cessation of polymorphonuclear leukocyte (PMN) infiltration, apoptosis of polymorphonuclear leukocytes at an inflammatory site (and subsequent efferocytosis by macrophages) and reprogramming of macrophages to a pro‐reparative phenotype. FPR2 agonists including lipoxins and ANXA1 have been demonstrated to drive all of these processes (Perretti & Godson, 2020). Hence, as FPR2 is a clear master‐receptor of resolution, ANXA1 pro‐resolving actions (as a result of relative preference for FPR2 over FPR1) are considered FPR2‐mediated.

To date, pro‐resolving agonists of FPR2 have been studied in various disease models, as well as in tissue healing (see Section 7). ANXA1‐derived peptides are one of the most well‐known endogenous FPR agonists (with actions evident at both FPR1 and FPR2) (Qin et al., 2015). It is thought that ANXA1 cleavage represents a catabolic event; cleavage of this protein has been reported in human bronchoalveolar lavage and blister exudates, suggesting this process may have biological significance, rather than an *in vitro* artefact (Tsao et al., 1998; Vong et al., 2007).

Notably, the ANXA1/FPR2 interaction accelerates tissue repair following biopsy‐induced mucosal epithelial wounding (Birkl et al., 2019; Leoni et al., 2015). Lipid mediators LXA_4_ and RvD1 stimulate regeneration of corneal epithelium via FPR2 (Kenchegowda et al., 2011; Zhang et al., 2018) and limit chronic allergic eye disease (Saban et al., 2019). Alleviated ROS accumulation and stimulation of antioxidant genes is a likely RvD1 mechanism‐of‐action, as these are reversed by subconjunctival WRWWWW (WRW4) administration either 24 h prior to, or 24 h after, removal of the corneal epithelium in diabetic mice (Zhang et al., 2018). Recently, ANXA1 was shown to stimulate the AMP‐activated protein kinase (AMPK) pathway via an FPR2‐dependent mechanism, promoting a pro‐reparative macrophage phenotype that facilitates skeletal muscle repair following injury (McArthur et al., 2020). The potential indications for FPR2‐targeted therapies are outlined in greater detail at Section 9. Whether FPR1 is also engaged in mechanisms of resolution of inflammation however remains to be established.

## 3 INTRACELLULAR SIGNALLING DOWNSTREAM OF FPR2: INSIGHTS FROM FPR LIGANDS

The FPR family canonically couples to the G_i/o_ subfamily of G proteins, as evidenced by the pertussis toxin (PTx)‐sensitivity of many FPR‐mediated effects in phagocytes. Signal transduction pathways triggered by formylpeptide analogues such as fMLF have been extensively characterised and shown to involve G_βɣ_‐mediated activation of PLC‐β and downstream PKC, release of intracellular Ca^2+^ stores and stimulation of PI3K‐Akt pathway (Selvatici et al., 2006; Ye et al., 2009). Recent developments, including phospho‐proteomics and subtype‐selective antagonists, have enabled greater interrogation of the signalling networks specifically downstream of FPR2 activation (Cattaneo et al., 2019).

FPR2 signalling modulates many critical intracellular functions including cell migration, proliferation, differentiation, apoptosis, intracellular communication and cell survival, with particular importance in immune cell function (Cattaneo et al., 2019; Selvatici et al., 2006). FPR2 stimulates both pro‐ and anti‐inflammatory signal transduction in both a ligand‐specific and cell‐background‐specific manner, such that the same ligand may promote seemingly opposing functional outcomes in different cell‐types (Cattaneo et al., 2013; Maddox et al., 1997; Perretti & Godson, 2020; Raabe et al., 2019). The underlying mechanism is still to be fully elucidated and may involve biased signalling or receptor dimerization (as explored in Section 4). The majority of FPR2‐mediated physiological effects appear to be downstream of G_i/o_ proteins, but there are also reports that the receptor couples to other G proteins (at least in heterologous expression systems), including G_q/11_, G_12_/G_13_ and G_15_/G_16_ (Badolato et al., 1995; Garcia et al., 2019; Jiang et al., 1996; Maddox et al., 1997; Ye et al., 2009). Interestingly, despite G_i/o_ being the canonical signalling partner for FPR2, the cAMP/PKA pathway does not appear to play a major role in FPR2 function; rather, most signalling is mediated by G_βɣ_ subunit activation of phospholipases.

Differential FPR2 signalling is dependent on type of ligand and cell‐background tested. LXA_4_ and its stable analogue aspirin‐triggered 15‐epi‐lipoxin A_4_ (ATL) inhibit or stimulate chemotaxis in human polymorphonuclear leukocytes and monocytes, respectively, with a transient rise in Ca^2+^ at therapeutic ligand concentrations observed only in monocytes (Chiang et al., 2006; Ge, Zhang, et al., 2020; Maddox et al., 1997). LXA_4_ also induces biphasic PLD activation in human polymorphonuclear leukocytes and HL‐60 cells and stimulates guanosine triphosphate hydrolase (GTPase) activity and release of esterified arachidonate in a pertussis toxin (PTx)‐sensitive manner in FPR2‐transfected CHO cells (Fiore et al., 1993, 1994). In human fibroblast‐like synoviocytes (FLS), LXA_4_ inhibits release of chemoattractant cytokine IL‐8 and up‐regulates release of tissue inhibitor of MMP (TIMP)‐2. In contrast, the pro‐inflammatory agonist serum amyloid A induces release of IL‐8 (CXCL8) and MMP3, and subsequent NF‐κB pathway activation, known to be important modulators of arthritis (Sodin‐Semrl et al., 2004). Serum amyloid A also stimulates PLC, phosphorylated ERK1/ ERK2 (pERK1/2), Ca^2+^ mobilisation and chemotaxis, via a G_βɣ_‐G_i/o_ mechanism, in human monocytes (Cattaneo et al., 2013).

The discovery of the potent FPR1/2 agonists, synthetic hexapeptides WKYMVm and its isomer WKYMVM (where Met^6^ is the D‐isomer of the amino acid in WKYMVm or L‐isomer in WKYMVM), shed further light on FPR2‐mediated signal transduction. WKYMVM/m stimulates Ca^2+^ mobilisation and neutrophil chemotaxis via tyrosine phosphorylation events. In human neutrophils, WKYMVM/m activates ERK1/2, JNK and PLA_2_ ‐mediated superoxide generation (Bae et al., 2003). In addition, WKYMVm elicits phosphorylation and membrane translocation of the p47^phox^ subunit of NADPH oxidase (required for NADPH oxidase‐dependent ROS generation) in IMR90 human fibroblasts (Ammendola et al., 2004; Bae et al., 2003). More recently, the AMPK pathway has been implicated in ANXA1/FPR2‐mediated macrophage polarisation toward an anti‐inflammatory tissue‐reparative phenotype in settings of muscle injury (McArthur et al., 2020).

It is widely recognised that GPCRs can also interact with β‐arrestins to elicit receptor desensitisation, internalisation and G‐protein‐independent signalling. The precise role of β‐arrestin1 and β‐arrestin2 in FPR2‐mediated signalling and endocytosis is still being established and may be pathway‐ and cell‐background‐dependent. In both mouse embryonic fibroblasts and HEK293 cells transfected with FPR2, β‐arrestin1/2 does not mediate ERK1/2 activation, but is required for FPR2 internalisation (Huet et al., 2007). In contrast, FPR2 agonists unable to recruit β‐arrestin show impaired chemotaxis in polymorphonuclear leukocytes, highlighting the importance of this pathway for cell migration (Gabl et al., 2017; Sundqvist et al., 2019). In addition, Sundqvist et al. pointed toward β‐arrestin‐independent FPR2 endocytosis, as the β‐arrestin/clathrin adaptor protein inhibitor barbadin was unable to inhibit endocytosis in FPR2‐overexpressing HEK cells, nor change agonist‐mediated FPR2 surface expression in human neutrophils (Sundqvist et al., 2020). The endogenous ligands RvD1 and LXA_4_ have also been proposed to recruit β‐arrestin, from data generated using DiscoverX's PathHunter enzyme fragment complementation technology that has a ProLink peptide fused to the FPR2 C‐terminus (Krishnamoorthy et al., 2010).

## 4 NOVEL PARADIGMS IN FPR2 SIGNALLING: BIAS AND BEYOND

Recent studies have explored the mechanisms underpinning the divergent signalling fingerprints, providing insight into the physiological and pathophysiological consequences of FPR2 biased agonism. Historically, the ternary complex model of receptor activation, which incorporates low‐ and high‐affinity ligand‐receptor interactions, as well as the pre‐coupled receptor interaction with an additional membrane component, has been used to describe GPCR activation (De Lean et al., 1980). Within this model, receptors can exist in two different conformations, ‘active’ and ‘inactive’. However, research over the past two decades has identified biased agonists that stimulate non‐uniform GPCR signal transduction, relative to a reference agonist acting at the same GPCR. Traditional analytical frameworks have been revisited to enable pharmacological analysis of biased agonism. An extension to the Black‐Leff operational model was developed to quantify the magnitude and enable statistical evaluation of, biased agonism, while correcting for the complexities of system and observational bias (Kenakin & Christopoulos, 2013). Such rigorous evaluation of experimentally observed biased agonism is important to inform drug discovery programs and provides a deeper understanding of GPCR signalling in (patho)physiology.

The FPR family of GPCRs is particularly amenable to biased agonism. FPR2 is recognised by numerous structurally diverse agonists (Table 1), both naturally occurring and synthetic, that mediate divergent cellular responses. Serum amyloid A is a pro‐inflammatory FPR2 agonist, stimulating chemotaxis of polymorphonuclear leukocytes and monocytes, promoting polymorphonuclear leukocytes adhesion and delaying neutrophil apoptosis (Dufton et al., 2010; El Kebir et al., 2007). In contrast, the FPR2 agonists LXA_4_, ANXA1, annexin 1‐(2‐26) and RvD1 stimulate anti‐inflammatory and pro‐resolving signalling, such as inhibition of polymorphonuclear leukocytes chemotaxis, recruitment and increasing macrophage efferocytosis (Dufton et al., 2010; Filep, 2013; Sansbury et al., 2020). Small molecules also mediate non‐uniform signalling fingerprints. Specifically, compound 17b has significant bias away from FPR1/2‐mediated Ca^2+^ mobilisation, relative to compound 43 (Qin et al., 2017). Importantly, the signal fingerprint observed for compound 17b, quantified using a heterologous expression system, was associated with superior *in vivo* cardioprotection following myocardial infarction in mice (Qin et al., 2017). Collectively, these studies demonstrate the therapeutic potential of FPR2 biased agonism, particularly for disorders associated with chronic inflammation.

In addition to signalling as a monomeric unit, FPR2 can form homodimers and heterodimers, a potential evolutionary mechanism to confer texture to agonist signalling in different cell‐types (Raabe et al., 2019). In experimental settings, FPR2 oligomerization imparts additional complexity to analysis of cellular responses, particularly in the context of biased agonism. Interestingly, the anti‐inflammatory and pro‐resolving agonists ANXA1, compound 43, Ac‐ANXA1 (2‐26) and LXA_4_, promote FPR1‐FPR2 heterodimerization. Whether such heterodimers reduce amount of FPR1 available for activation by pro‐inflammatory agonists represents a contributing mechanism by which FPR2 anti‐inflammatory agonists reduce inflammatory signalling remains to be determined. In contrast, the pro‐inflammatory agonist serum amyloid A and the FPR antagonists cyclosporin H (CsH) and synthetic peptide Trp‐Arg‐Trp‐Trp‐Trp‐Trp‐NH_2_ (WRW4) have no significant effect on FPR1‐FPR2 heterodimerization. Similarly, FPR2 homodimerization is enhanced in the presence of ANXA1 and annexin 1‐(2‐26), but decreased in the presence of serum amyloid A. Initial studies suggest a link between the formation of FPR2 oligomers and downstream phosphorylation of p38MAPK and the small heat‐shock protein (Hsp27), a pathway stimulated by ANXA1, but not serum amyloid A, and associated with the release of the anti‐inflammatory mediator IL‐10 (Cooray et al., 2013). Biphasic modulation of FPR2 activation in the presence of aspirin‐triggered 15‐epi‐lipoxin A_4_ (ATL; Ge, Zhang, et al., 2020), Ac‐ANXA1 (2‐26) and amyloid beta 42 (Zhang, Gong, et al., 2020) supports the notion of multiple allosteric ligand binding sites, either within the monomeric unit or across an FPR2 dimer.

The molecular mechanisms underpinning FPR2 biased agonism remain to be fully elucidated. GPCR biased agonism typically involves agonist‐specific stabilisation of a unique spectrum of receptor conformations, which in turn promote differential recruitment of intracellular effector proteins. As discussed above, activation of FPR2 preferentially couples to G_i/o_ proteins and recruits β‐arrestin. Moreover, FPR2 can also promote G_q/11_ protein signalling and was recently shown to form complexes with G_12_ proteins, but did not promote G_12_ activation, thereby inhibiting the recruitment of G_12_ proteins to other GPCRs (Okashah et al., 2020; Park et al., 2020). Structure–function studies suggest differential signalling may be associated with specific receptor domains, with structurally diverse agonists recognising spatially distinct FPR2 binding sites (Le et al., 2005). For example, transmembrane (TM) domain 6 and extracellular loop (ECL) 3 appear to have an important role in FPR2‐mediated chemotactic signalling (Le et al., 2005). In contrast, ANXA1 signalling is dependent on the FPR2 N‐terminal region and ECL2, whereas binding of compound 43 involves TM3 (Bena et al., 2012). Structural insights into FPR2 agonist engagement are considered in detail below.

## 5 STRUCTURAL INSIGHTS INTO AGONIST ENGAGEMENT OF FPR2

Our understanding of the way FPR2 can be differently regulated by agonists has increased by elucidation of FPR2 structures, molecular modelling, molecular dynamics and mutagenesis studies (Chen et al., 2020; Zhuang et al., 2020). In 2020, two structures were determined of FPR2 bound to the agonist WKYMVm. The first structure was determined using cryo‐electron microscopy and was of the agonist bound receptor in complex with the inhibitory heterotrimeric G protein, G_i1_β_1_γ_2_ (Zhuang et al., 2020), whereas the second structure was determined in the absence of the G protein, using x‐ray crystallography (Chen et al., 2020). Regardless of the presence or absence of the G protein, the FPR transmembrane bundle was in an active state conformation, with similar peptide interactions observed in both structures (Chen et al., 2020; Zhuang et al., 2020). More recently, three additional cryo‐EM structures have emerged of FPR2‐G_i1_β_1_γ_2_ complexes with either the formylpeptide fMLFII, synthetic anti‐inflammatory peptide CGEN‐855A or the non‐peptide compound 43 bound (Zhuang et al., 2022). Combined with molecular modelling, receptor mutagenesis and peptide structure activity relationship studies, these structures provide detailed molecular insights into how different agonists bind and activate FPR2.

**FIGURE 2:**
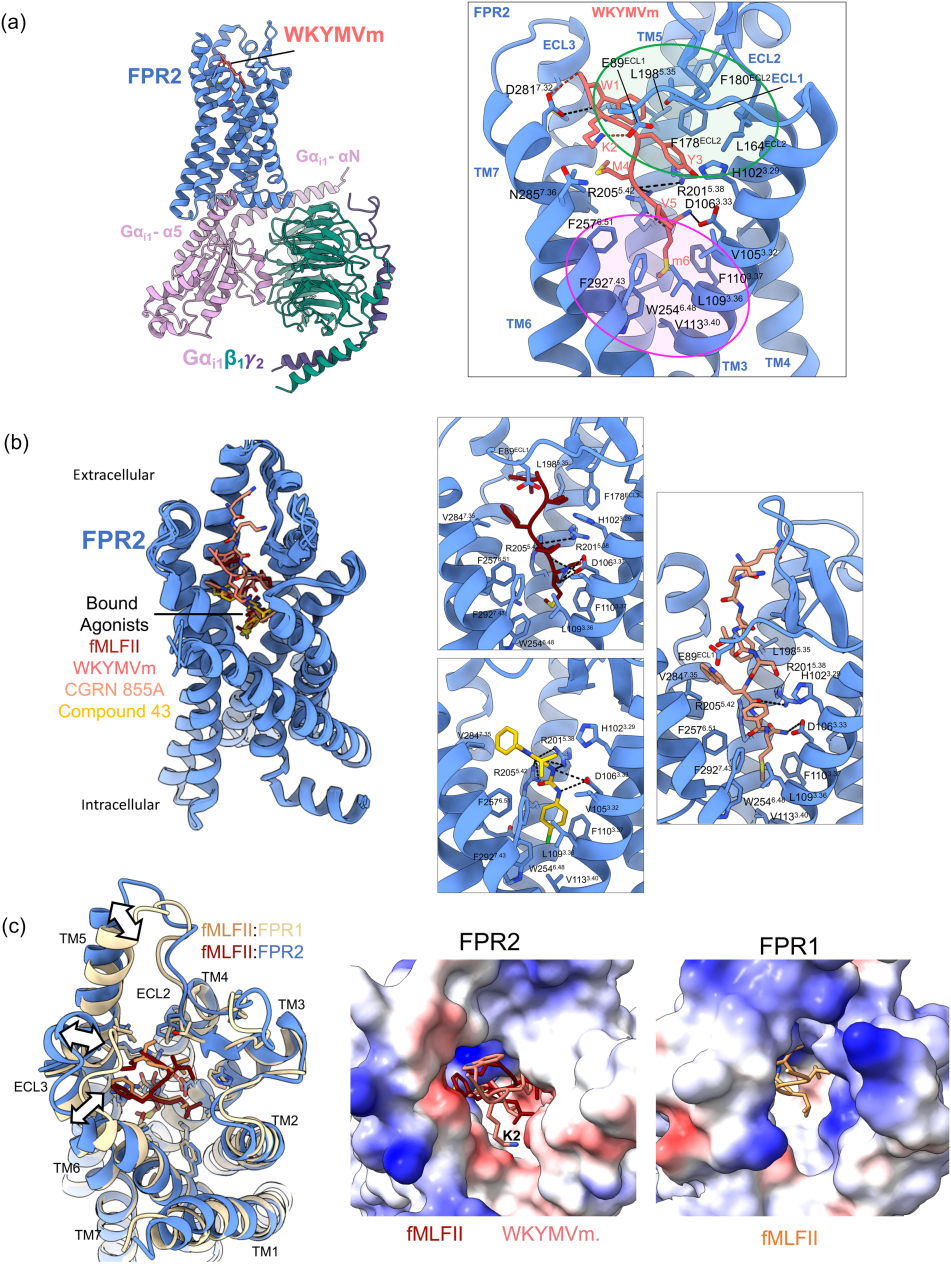
Agonist bound FPR2‐G_i1_ and FPR1‐G_i1_ complexes. (a) Left: the first FPR2 cryo‐EM structure (PDB:6OMM) bound to the peptide agonist WKYMVm and the heterotrimeric Gi_1_ protein. The large inset on the right shows a close up of the WKYMVm binding site, with interacting FPR2 residues shown. H‐bonds and salt bridges between the receptor and the peptide are shown as black and red dashed lines, respectively. The regions highlighted in the green and pink circles show the hydrophobic clusters of residues at the top and bottom of the binding pocket. (b) Left: overlay of four agonist bound FPR2 cryo‐EM structures (PDBs: 6OMM, 7T6V, 7T6U, 7T6S) reveals different agonists bind in overlapping poses and induces a similar FPR2 conformation. Right insets show a close up of the binding sites of fMLFII (top left), compound 43 (bottom left) and CGEN 855 with interacting FPR2 residues shown. (c) Left: overlay of the cryo‐EM structures of FPR1 (PDB:7T6T) and FPR2 (PDB:7T6V) shows similar binding poses of fMLFII in both receptors. White arrows highlight differences in the conformation of the two receptors when fMLFII is bound. Middle and right: surface representation coloured by electrostatic charge (blue, positively charged; white, neutral; red, negatively charged) of the FPR2 (middle, with fMLFII and WKYMVm bound) and FPR1 (right, with fMLFII bound) looking down from the extracellular surface into the peptide binding pocket. This highlights the large open and negatively charged FPR2 binding pocket, relative to the more closed, positively charged FPR1 binding cavity. The top of the FPR2 binding pocket that accommodates the side chain of lysine 2 (K2) of WKYMVm is predominantly negatively charged, providing a rationale for the preference of for positively charged over negatively charged residues within the C‐terminus of FPR2 peptide agonists.

The FPR2 binding pocket is amphiphilic and consists of two main hydrophobic clusters (Figure 2) where fMLFII and WKYMVm bind with overlapping poses. This binding cavity is bordered by TMs 3, 5 and 7, as well as ECLs 1–3, with both peptides penetrating relatively deep within the TM core, adopting an extended conformation (Figure 2). The FPR2 binding pocket is amphiphilic and consists of two main hydrophobic clusters (Figure 2) with the N‐terminus of the formylpeptide interacting in a similar manner to the C‐terminus of WKYMVm (Chen et al., 2020; Zhuang et al., 2022). The hydrophobic cluster located at the top of the binding cavity accommodates the peptide N‐terminal aromatic W1 and Y3 of WKYMVm, and the C‐terminal FII of fMLFII, while the second cluster, located at the base of the binding pocket, forms interactions with the C‐terminal V5 and D‐Met6 of WKYMVm, and the N‐terminal fM1 and L2 of fMLFII (Figure 2). Alanine mutagenesis studies support the importance of residues in these hydrophobic clusters in agonist affinity and efficacy (Chen et al., 2020). These interactions are also consistent with previous structure–activity studies on the hexapeptide MKYMPM‐NH_2_ (an FPR2 agonist related to WKYMVm), which identified essential roles for hydrophobic residues at positions 3, 5 and 6 in hexapeptide agonist activity (Seo et al., 1997).

In addition to hydrophobic interactions, fMLFII and WKYMVm also form extensive polar interactions with the base of the FPR2 binding cavity with three polar residues, D106^3.33^, R201^5.38^ and R205^5.42^ (subscript refers to Ballesteros‐Weinstein nomenclature; Ballesteros & Weinstein, 1995; Chen et al., 2020; Zhuang et al., 2020). Specifically, Y3 and the acetamide group of D‐Met form an extensive polar network with D106^3.33^, R201^5.38^ and R205^5.42^, and these receptor residues also participate in hydrogen bond interactions with the backbone of M4 and V5. The N‐formyl group of fMLFII forms similar interactions to the acetylamide group of the D‐Met6 of WKYMVm, forming a hydrogen bond with R201^5.38^, while FPR2 residue R201^5.38^ also hydrogen bonds with the backbone carbonyl of L2 of the peptide and D106^3.33^ with the main chain amine groups of fM1 and L2 of fMLFII (Figure 2). Alanine mutations of these receptor residues cause a dramatic decrease in both fMLFII‐ and WKYMVm‐induced FPR2 activation and Gi coupling, supporting their critical role for the agonism of both peptide ligands (Chen et al., 2020; Zhuang et al., 2020). In addition, substitution of m6 with L‐Met reduces affinity and efficacy by 20–100‐fold, likely by disruption of the C‐terminal hydrogen bonding as a result of a rotation in the C‐terminal amide (Chen et al., 2020).

Unlike formylpeptides, the N‐terminus of WKYMVm is also stabilised by two salt bridges between D281^7.42^ and the N‐terminal amide, and E89^ECL1^ and K2. In the X‐ray structure, there is also an additional hydrogen bond positioned between N285^7.46^ and K2. Alanine mutagenesis of D281^7.42^, E89^ECL1^ and N285^7.46^ had less dramatic effects than those deeper in the pocket (D106^3.33^, R201^5.38^ and R205^5.42^), suggesting these N‐terminal interactions are less critical for the agonist function of WKYMVm (Chen et al., 2020).

The interaction mode of the formylated methionine of fMLFII deep within the FPR2 cavity can explain selectivity of FPR2 for different formylpeptides, where the composition of the C‐terminal amino‐acids determines their optimal interaction (He et al., 2014). FPR2 prefers pentapeptide formylpeptides, such as fMLFII and fMLFIK, over shorter tri and tetra formylpeptides, such as fMLF and fMLFW (He et al., 2014; Ye et al., 2009). In addition, peptides with negatively charged amino‐acids at the C‐terminus have only weak affinity for the receptor. Docking studies have revealed the C‐terminal carboxyl group of short formylpeptides, such as fMLF, is located in a negatively charged environment due to the presence of E89 and D281^7.32^, which is energetically unfavourable (Chen et al., 2020). This negatively charged environment also favours positively charged residues at the C‐terminus and can explain the 500–5000‐fold higher preference of FPR2 for fMLFK over fMLFE with K4 able to form a salt bridge with E89^ECL1^ or D281^7.32^, whereas these residues are repulsive with E5 (Chen et al., 2020; He et al., 2014). The fMLFII‐bound FPR2 structure and previous docking studies can also rationalise the preference of FPR2 for pentapeptides fMLFII and fMLIK, where the two isoleucines in fMLFII reside in a similar binding pocket to W1 in WKYMVm and form extensive hydrophobic interactions with residues in ECL2, ECL3, TM5 and TM6. FMLFIK also forms these similar hydrophobic interactions; however, K5 can also hydrogen bond with D281^7.32^ (Chen et al., 2020; Zhuang et al., 2020). D281^7.32^G leads to decreased affinity of both fMLFK and fMLFIK for FPR2 (He et al., 2014) and supports the role of this residue in the selectivity of FPR2 for positively charged C‐terminal residues.

The FPR2 cryo‐EM structure is open at the extracellular face, creating a large pocket that can accommodate larger peptides and the amphiphilic nature of the binding pocket provides an environment suitable for the binding of lipid molecules. In addition, molecular dynamics simulations on FPR2 in the absence of a bound ligand and G protein revealed the receptor extracellular region is highly flexible and this may be important in allowing the receptor to recognise chemically diverse ligands (Zhuang et al., 2020), for which FPR2 is renowned. This is supported by the recent cryo‐EM structure of FPR2 bound to CGEN‐855, which a 21 amino‐acid peptide selective for FPR2 that interacts with its C‐terminal amidated methionine residue deep within the receptor binding cavity (Zhuang et al., 2022). Similar to WKYMVm, the C‐terminal amidated M21 of CGEN‐855A forms hydrophobic interactions with L109^3.36^, F110^3.37^, V113^3.40^ and W254^6.48^ and polar interactions with D106^3.33^ and R205^5.42^, while R201^5.38^ also forms a hydrogen bond with the backbone of the peptide C‐terminus. While residues 14–20 of CGEN‐855A form extensive hydrophobic interactions with FPR2 binding site (Figure 2), density for the remainder of the peptide was not observed in the cryo‐EM map, suggesting the N‐terminal 13 residues of the peptide does not form extensive interactions with the receptor (Zhuang et al., 2022).

While there are currently no experimentally determined structures, molecular docking of LXA_4_ into the FPR2 structure suggests this molecule occupies a shallow binding pocket deep within the receptor core that differs from the binding site of the agonists in the experimentally determined structures (Ge, Liao, et al., 2020). Nonetheless, the binding pose partially overlaps the mid/C‐terminal‐region (YMV and MLF) of WKYMVm and fMLFII, forming interactions with a number of FPR2 residues that also interact with these peptides, including hydrogen bonding with D106^3.33^, R201^5.38^ and R205^5.42^, and Van der Waal interactions with W254^6.48^. This suggests these residues may be critical for recognition of diverse agonists by FPR2 and for triggering receptor activation.

The recent cryo‐EM structure of compound 43 bound to FPR2 provided the first structural insights into how to target FPR2 with non‐peptide agonists (Zhuang et al., 2022). Compound 43 binds at the base of the FPR2 binding pocket with the chlorophenyl group occupying the narrow cavity in a similar manner to the terminal methionine of the peptide agonists. Similar to peptide agonists, the chlorophenyl group forms extensive van der Waal interactions with residues within the hydrophobic cluster at the base of the binding pocket, including with FPR2 residues L109^3.36^, F110^3.37^, V113^3.40^, W254^6.48^, F257^6.51^ and F292^7.43^ (Figure 2). The urea and pyrazole moieties of the compound form polar interactions with D106^3.33,^ R201^5.38^ and R205^5.42^, while the pyrazole group also forms van der Waal interactions with residues in TMs 2, 3 and 7 (Figure 2). Mutagenesis of these polar residues dramatically reduces the agonism of compound 43, in a similar manner to observe for peptide agonists, suggesting formation of polar interactions with these residues is crucial for agonist activity of FPR2 ligands.

Compared with other chemoattractant GPCRs that bind peptide ligands, FPR2 agonists insert more deeply into the receptor core, allowing direct contacts V113^3.40^ and W254^6.48^, key residues within the conserved PIF (P^5.50^, I^3.40^ and F^6.44^) and toggle switch motifs, respectively, which are important for activation of class A GPCRs (Deupi & Standfuss, 2011; Zhuang et al., 2020, 2022). In the docking study by Ge et al., LXA_4_ also formed direct interactions with W254^6.48^ (Ge, Zhang, et al., 2020). These interactions may induce conformational changes in these ‘transmission’ motifs through steric effects, enabling receptor activation. Indeed, the outward conformation of TM6 in the X‐ray structure was identical to the cryo‐EM structure of FPR2 bound to G_i_ (Chen et al., 2020; Zhuang et al., 2020). This is in contrast to many class A GPCRs, where ligands do not directly engage the transmission switch residues and a full outward movement of TM6 is not always observed in structures of agonist bound class A GPCRs in the absence of a transducer occupying the intracellular binding cavity. The polar interactions formed by all agonists with D106^3.33^, R201^5.38^ and R205^5.42^ of FPR2 are proposed to correctly position the terminal methionine (peptides) or the chlorophenyl group (compound 43) into the narrow cavity above V113^3.40^ and W254^6.48^. This deep ligand binding pocket may allow easy access to the transmission switch that also enables diverse ligands to readily activate the receptor. Regardless of the bound agonist, the conformation of the active state of the receptor is identical in each of the cryo‐EM structures determined to date, where, at the intracellular face, activated FPR2 interacts predominantly with the α5 helix of the Gα_i_ subunit of the heterotrimeric G protein, which inserts into the cavity at the cytoplasmic domain of FPR2 and forms both polar and hydrophobic contacts. Additional hydrophobic interactions occur between TM4, TM5, TM6, intracellular loop (ICL) 3 and intracellular loop 2 of FPR2 with other regions within Gα_i_ and between FPR2 helix 8 and the G_β_ subunit (Zhuang et al., 2020, 2022).

In addition to cryo‐EM structures of FPR2, there is also a cryo‐EM structure of FPR1 bound to fMLFII and Gi (Zhuang et al., 2022). While the interactions formed by the N‐terminal formyl‐Met of fMLFII with FPR1 were similar to FPR2, two sequence differences in the FPR1 and FPR2 peptide binding site can explain the higher affinity of fMLFII for FPR1. F257^6.51^ and H103^3.29^ in FPR2 are replaced by Y257^2.61^ and F102^3.29^ in FPR1, enabling FPR1 to form an additional hydrogen bond and van der Waal interactions with fMLFII that are not formed by FPR2. Mutation of these residues to the equivalent residues in FPR2 reduces the affinity of fMLFII for FPR1. In addition, F3‐I5 of fMLFII forms more extensive hydrophobic interactions with FPR1 than FPR2 and the extracellular loops close in to generate a narrower binding cavity. While the relatively wide binding pocket identified in the FPR2 structures revealed from cryo‐EM studies are suitable for binding long peptides, proteins and lipid molecules (Chen et al., 2020), the narrower cavity for FPR1 would restrict the binding of longer peptides and can explain the higher affinity of these agonists for FPR2 over FPR1. In contrast to the longer formylpeptides preferred by FPR2, FPR1 can also be activated by shorter formylpeptides, such as fMLF. The differential selectivity of FPR1 and FPR2 for short formylpeptides has been attributed to the differential charge distribution at the top of the binding pocket. Within this region, FPR1 has positive charge distribution amenable to accommodating the formylpeptide C‐terminal carboxyl group, whereas FPR2 has a negative charge environment (Figure 2), making the binding of short formylpeptides energetically unfavourable (Chen et al., 2020). While the recent determination of FPR2 structures provide some mechanistic insights into FPR2 agonist recognition patterns, receptor activation and Gi protein coupling, additional structural and pharmacological studies with other ligands, including lipids, are required, to better understand how FPR2 recognises diverse chemical ligands that can generate distinct, and opposite, roles in inflammation and disorders that are a consequence of inflammation and dysregulated resolution. A greater understanding of the binding mode of structurally distinct FPR2 ligand and subsequent receptor–effector interactions that engage proinflammatory and pro‐resolving signal transduction, will facilitate the rational design of FPR2 biased agonists in drug discovery.

## 6 PATHOPHYSIOLOGICAL SIGNIFICANCE OF ALTERED FPR2 EXPRESSION: FROM GENE MANIPULATION TO TRANSCRIPTOMICS

FPR1, FPR2 and FPR3 are expressed in both myeloid and non‐myeloid cells. In humans, FPR2 is ubiquitously expressed at relatively low levels, including in immune cell populations, as well as endothelial, epithelial and neuronal cells (Becker et al., 1998; Migeotte et al., 2006). Given the diversity of endogenous FPR2 ligands that can elicit pro‐resolving or pro‐inflammatory signalling cascades, understanding the pathophysiological significance of FPR2 expression is both challenging and intriguing. This question has been investigated by *FPR2* gene manipulation studies. Seven mouse *Fpr* (lower case after the first letter denotes rodent genes) have been identified, with several described as orthologs of human *FPR2* (Ye et al., 2009). Gene manipulation strategies typically involve either murine *Fpr2/3* knockout (i.e. double knockout of the two homologues of human *FPR2*) or transgenic overexpression of human FPR2. For example, myeloid‐selective overexpression of human FPR2 using a human CD11b promoter led to reduced neutrophil infiltration in a mouse model of zymosan‐induced peritonitis (Devchand et al., 2003). In keeping with this, in murine models of acute inflammation (ischaemia–reperfusion insult, carrageenan‐induced paw oedema, pneumococcal meningitis and liver injury), genetic deletion of murine *Fpr1/2* leads to a more severe inflammatory phenotype (Dufton et al., 2010; Giebeler et al., 2014; Oldekamp et al., 2014). A role for *Fpr2* in wound healing has also been reported, with mice deficient in *Fpr2* displaying impaired re‐epithelialization during skin injury (Hellmann et al., 2018). Congruently, aptamers acting as FPR2 agonists are associated with enhanced wound healing in *in vitro* models (Arriba et al., 2022).

In the context of human health, it is suggested that genetic variations in *FPR2* may play a role in disease susceptibility and responsiveness in specific human pathologies. A single nucleotide polymorphism (SNP) in the *FPR2* promoter (A > G 220), associated with reduced *FPR2* gene (10‐fold) and protein (3‐fold) expression, was identified from genotyping polymorphonuclear leukocytes from 232 subjects, detected in 1 of 132 patients under 55 years old with prior myocardial infarction, but not in 100 healthy individuals (Simiele et al., 2012). Likewise, a different single nucleotide polymorphism (rs11666254; A > G) in the promoter region of *FPR2* (also associated with reduced receptor expression) was associated with increased susceptibility of patients (*n* = 646) to develop sepsis after severe trauma (Zhang, Gao, et al., 2020). Here, the GA or AA genotype was associated with a significantly higher risk of developing sepsis than the GG genotype (GA vs. GG OR 1.806, 95% CI 1.176–2.773, *P* = 0.007; AA vs. GG OR 3.009, 95% CI 1.788–5.062, *P* = 0.000). Conversely, a potentially beneficial intronic single nucleotide polymorphism (rs1769490; T > G) is associated with higher peripheral blood mononuclear cells (PBMC) expression of FPR2 and reduced risk of aspirin hypersensitivity in asthmatics (Kim et al., 2012; Zhang, Lu, et al., 2017). While these studies provide suggestive evidence for FPR2 polymorphisms impacting on expression and human disease susceptibility, it is acknowledged that patient cohort sizes are relatively small. Future efforts require significantly larger cohorts, deep‐sequencing approaches to investigate rare variants and replication studies across different ethnicities. Several studies have investigated the role of FPR2 in a range of human diseases. For example, up‐regulation of FPR2 has been reported in atherosclerotic lesions, ovarian cancer and inflamed synovial tissues compared with levels in healthy control tissue (Brennan, Mohan, McClelland, de Gaetano, et al., 2018; O'Hara et al., 2004; Petri, Laguna‐Fernandez, Tseng, et al., 2015; Xie et al., 2017). In murine macrophages, transcriptional profiling in either undifferentiated (M0), M1‐like or M2‐like conditions, identified an up‐regulation of the mouse FPR2 expression in M1 macrophages versus M0 and M2 cells (Jablonski et al., 2015) However, the opposite has also been described, with FPR2 expression reported to be reduced in human abdominal aortic aneurysm lesions compared with levels in health controls (Petri et al., 2018). Decreased FPR2 expression has also been observed in patients with asthma versus matched healthy individuals (Planaguma et al., 2008). These contrasting findings raise several possibilities regarding the central role of this receptor in disease pathogenesis. Considering the range of pro‐ and anti‐inflammatory agonist acting on FPR2, downstream effects may be highly impacted by different stimuli and the cell‐types investigated. It is plausible that in certain disease settings, an increase in FPR2 expression reflects the presence of infiltrating FPR2‐expressing immune cells to the site of an inflammatory response. From a pro‐resolving pharmacotherapy perspective, any increase in FPR2 receptor availability is advantageous when considering the exploitation of FPR2 agonists as therapeutics. In contrast to this, the observations of reduced FPR2 expression in human disease support the anti‐inflammatory, pro‐resolution function of FPR2, the impairment of which may represent an underlying pathogenetic mechanism of inflammatory chronic diseases. Differences in disease settings and tissue sampling conditions may explain some of these conflicting results, together with the balance between physio‐pharmacology versus pathology. Strategies aiming at augmenting FPR2 expression levels in diseased tissues could also be of therapeutic value. Going forward, detailed single‐cell and spatial transcriptomic profiling of inflamed tissues will be required to clarify the precise cell‐specific changes in FPR2 expression in human disease. Furthermore, development of cell‐specific *Fpr2* overexpression/knockout murine models will be an essential tool to understand the biological consequences of manipulating *Fpr2* expression.

While there is great interest in elucidating the downstream effector pathways of FPR2 agonism, the upstream molecular cues that control FPR2 transcription and translation during inflammation initiation and resolution remain poorly characterised, with a limited number of pro‐ and anti‐inflammatory regulators identified. In fibroblast‐like synoviocytes, proinflammatory TNF‐α, but not IL‐1β or IL‐6, up‐regulated FPR2 expression (O'Hara et al., 2004). Enterocytes exposed to a panel of inflammatory cytokines (IL‐1β, 1L‐4, IL‐6, IL‐13, IFN‐γ) up‐regulated FPR2 expression in response to all stimuli within 48 h, with IL‐13 and IFN‐γ identified as potent transcriptional activators (Gronert et al., 1998). Another cytokine, IL‐10, has been identified as both a transcriptional activator and downstream effector of FPR2 (Cooray et al., 2013; Gronert et al., 1998; Locatelli et al., 2014). It is clear from these data that FPR2 expression may be regulated by pro‐ and anti‐inflammatory stimuli and that the response to stimulation depends on the cell‐type investigated.

Analysis of the *FPR2* gene promoter has identified several functionally relevant sequence motifs, including a binding site for the Sp1 transcription factor critical for FPR2 promoter activity (Simiele et al., 2012). Further characterisation of the FPR2 promoter in monocytes and macrophages identified Oct1 and Sp1 as key transcription factors regulating FPR2 expression (Waechter et al., 2012). Epigenetic regulatory mechanisms at the *FPR2* gene have also been described, which may in part explain how *FPR2* gene expression is controlled in a cell‐specific manner (Simiele et al., 2016). Here, chromatin immunoprecipitation (ChIP) analysis of FPR2 in cells expressing high and low levels of FPR2 provided evidence that epigenetic silencing at FPR2 is mediated via H3 lysine patterns. Specifically, in polymorphonuclear leukocytes expressing abundant levels of FPR2, this was characterised by high levels of the transcriptionally permissive marks H3K27ac and H3K4me3 and low levels of the transcriptionally repressive mark H3K27me3. The opposite pattern was observed in breast cancer MDA‐MB231 cells that express relatively low FPR2 levels.

The role of non‐coding RNA in regulating FPR2 expression has also been explored (Fan et al., 2019; Pierdomenico et al., 2015), with multiple putative microRNA (miRNA) binding sites identified in the 3′‐UTR of FPR2. Among these, there is evidence for a direct interaction between microRNA miR‐181b and FPR2 in human macrophages, with a reduction in miR‐181b levels leading to FPR2 up‐regulation during monocyte‐to‐macrophage differentiation (Pierdomenico et al., 2015). The miR‐181b‐FPR2 axis was investigated further in airway epithelial and monocyte‐derived macrophage cells from cystic fibrosis patients, with evidence that miR‐181b is overexpressed whereas FPR2 is down‐regulated, in cystic fibrosis versus healthy cells. Anti‐miR‐181b strategies in these cells restored FPR2 expression, highlighting a potentially novel therapeutic target in cystic fibrosis (Pierdomenico et al., 2017).

With recent advances in gene‐ and protein‐profiling techniques, it is now possible to investigate global pathway responses downstream of FPR2. In this regard, studies have determined the transcriptome responses following exposure to FPR2 agonists. For example, in the diabetic apolipoprotein (ApoE)^−/−^ mouse, RNA‐seq profiling of mouse kidneys in animals administered endogenous LXA_4_ or a synthetic mimetic (Benzo‐LXA_4_) identified subsets of transcripts significantly changed in diabetic kidneys. In this study, pathway analysis identified established (TGF‐β1, PDGF, TNF‐α and NF‐κB) and novel (early growth response‐1 [EGR‐1]) networks activated in diabetes and regulated by lipooxins (LXs; Brennan, Mohan, McClelland, Tikellis, et al., 2018). Transcriptome profiling of murine bone marrow‐derived macrophages stimulated with the FPR2 agonist RvD1 identified 627 differentially regulated transcripts enriched for vascularization, immunity and host defence‐related pathways (Sansbury et al., 2020). Several miR‐mediated regulatory networks have also been identified downstream of FPR2 agonism, including LXA_4_‐mediated up‐regulation of lethal‐7 (let‐7) and mir‐126‐5p gene expression (Brennan et al., 2013, 2017; Codagnone et al., 2017) and RvD1‐mediated regulation of miR‐21, miR‐146b, miR‐219 and miR‐155 (Rajasagi et al., 2017; Recchiuti et al., 2011). Beyond RNA, the global phospho‐signalling response following FPR2 activation has also been explored by MS/MS in lung cancer CaLu‐6 cells exposed to the synthetic agonist WKYMVm, identifying 290 differentially phosphorylated proteins, with an enrichment for cell cycle and apoptosis‐related proteins (Cattaneo et al., 2019).

## 7 FPR2 AND OTHER PRO‐RESOLVING RECEPTORS

FPR2 was the first GCPR reported to be activated by both lipid (e.g. LXA_4_) and peptide/protein mediators (e.g. ANXA1) (Perretti et al., 2002). Over time, FPR2 has also been identified as the receptor mediating the actions of omega‐3 derived RvD1. Considering the ability of FPR2 to convey cell signals from cell activators, as well as *tout‐court* pro‐inflammatory agonists like serum amyloid A (He et al., 2003), the nature of FPR2 has been debated for quite a while. Is it a pro‐resolving or a pro‐inflammatory receptor and, as such, do we need to develop agonists or antagonists to it? The generation of the mouse nullified for the orthologues of human FPR2, shed some light. In 2010, two different mouse colonies were described: ‐ one from Rod Flower and his team (Dufton et al., 2010), the other by Ji Ming Wang and colleagues (Chen et al., 2010). At first sight, even these tools—at the time considered highly innovative—were unable to help clarify the biology, as in one case, *Fpr2* null mice displayed higher degree of inflammation of the joints and peritoneal cavity, while in the other, a lower degree of pulmonary inflammation was evident. However, further work indicated the overall protective nature of FPR2, also considering the life‐saving role of polymorphonuclear leukocyte infiltration and of inflammation in more general terms. The specific time and location can impact on the outcome downstream of FPR2 activation or absence of the receptor. Nonetheless, cases have been reported where absence of FPR2 delays the onset of the experimental disease, as in atherosclerosis progression (Petri, Laguna‐Fernandez, Gonzalez‐Diez, et al., 2015).

The chemerin receptor 1 (Chemerin_1_; ChemR23) is probably one of the first receptors associated with resolution, transducing the properties of each of resolvin (RvE1; Arita et al., 2007), the pro‐inflammatory protein chemerin (Hart & Greaves, 2010) and its bioactive peptide, chemerin15 (Cash et al., 2008, 2013), although the latter is anti‐inflammatory and tissue‐protective. The phenotype of ChemR23‐null mice is not particularly remarkable, displaying only mild obesity in the absence of unchecked adipose inflammation (Rouger et al., 2013). Interestingly, a novel monoclonal antibody (mAb) that activates ChemR23 in a biased fashion (mimicking RvE1, but not chemerin, signalling) was shown to accelerate resolution and counteract ongoing inflammation and tissue fibrosis, in animals subjected to experimental colitis (Trilleaud et al., 2021). This innovative therapeutic could open similar strategies for FPR2 and other pro‐resolving receptors.

When RvD1 was added to the list of FPR2 agonists, it was also noted that this mediator could activate the orphan receptor GPR32 (Krishnamoorthy et al., 2010). Interestingly, the latter does not have a murine counterpart, making it challenging to GPR32 in experimental settings of disease. Studies with human cells and tissues have identified GPR32 engagement with RvD1, in some cases with higher affinity than FPR2 (Norling et al., 2012). GPR32 is expressed by vascular cells and could mediate vasculoprotective actions of resolvins in atherosclerosis and vascular stiffening (Pirault & Back, 2018). Absence of GPR32 is associated with chronic heart failure and could explain lack of response to RvD1 (Chiurchiu et al., 2019). Modulation of human macrophage responses has also been reported, with GPR32 promoting a pro‐resolving phenotype (Schmid et al., 2016). Further, a recent elegant study revealed an atheroprotective role of GPR32 in a transgenic mouse colony lacking endogenous FPR2, a feature associated with modulation of leukocyte recruitment and macrophage efferocytosis (Arnardottir et al., 2021; Mena & Spite, 2021).

While FPR2 is so dominant in the spectrum of pro‐resolving receptors, nonetheless, other pro‐resolving GPCRs have been identified over the past few years and these could represent important targets for drug discovery (although typically deletion of receptor expression is less convincing than is the case for FPR2). GPR18 (G protein‐coupled receptor 18) was identified as the receptor for resolvin D2 (RvD2) through GPCR screening (Chiang et al., 2015) and was reported to have modulatory roles on CD8 T‐cell recruitment in the small intestine, as this was attenuated in mice nullified for the receptor (Wang et al., 2014). In the context of resolution, RvD2 is a potent regulator of leukocyte responses in experimental sepsis (Spite et al., 2009) and these effects are lost in GPR18‐knockout mice (Chiang et al., 2017). RvD2/GPR18 promotion of bacteria phagocytosis is pivotal to these attributions, a fundamental process in the cascade of events that characterise the resolution of inflammation. GPR18 has also been implicated in other pathophysiological settings evolving around the central nervous system and metabolic status. In some contexts, GPR18 antagonism may be a potential way forward for weight loss (Kotanska et al., 2021). Among the pro‐resolving receptors discussed here, leucine‐rich‐repeat‐containing G protein‐coupled receptor 6 (LGR6) is one of the most recent identified, mediating the properties of maresin‐1 (Chiang et al., 2019), again with a focus on phagocyte reactivity. Modulation of macrophage reactivity by maresin‐1 through LRG6 has also been reported in experimental aorta aneurism (Elder et al., 2021). Side‐by‐side comparison of FPR2 and GPR18 in wound‐healing revealed a dominant role for the former in pathological settings (FPR2 knockout animals displayed a stronger phenotype), while agonist application at either promoted keratinocyte migration (Hellmann et al., 2018).

The existence of a circuit between two FPR2 agonists, ANXA1 and LXA_4_, has also been reported in more complex settings, such as those of obesity induced adipose inflammation, where chronic administration of LXA_4_ exerted tissue protection via switching macrophage phenotype while at the same time increasing tissue expression of ANXA1 (Borgeson et al., 2015). It is therefore a little surprising that animals lacking FPR2 display marked macroscopic effects, such as lethality, in experimental sepsis (Gobbetti et al., 2014). FPR2 is central to several of the fundamental mechanisms of resolution and it can link pro‐resolving agonists to their protective responses; therefore, its absence (or malfunction) represents a non‐redundant pathological situation that exacerbates the host response leading to an unbridled and often self‐harming reaction. As discussed in Section 6, analyses of human databases have identified mutations for FPR2 in diseases of the cardiovascular system.

In summary, the powerful biological properties exerted in multiple experimental settings by FPR2 agonists, together with the clear phenotype of *Fpr2* null mice and the initial work in human genomics linking the receptor to diseases status, as presented in the previous section, justify the pharmacological exploitation of this receptor to kickstart resolution pharmacology.

## 8 FPR2 AGONISTS: VIEW TO THE FUTURE

### 8.1 FPR2 modulators under preclinical and clinical development

There is growing academic and industry interest in discovering and developing FPR2 agonists as novel therapeutic approach for inflammatory disease, as reviewed recently (Maciuszek et al., 2021). Given that endogenously produced specialised pro‐resolving mediators can be rapidly metabolised (e.g. by prostaglandin dehydrogenase), their therapeutic potential may be somewhat limited. Significant efforts have been made to develop more stable, specialised pro‐resolving mediators (SPMs, e.g. LXA_4_ and ANXA1), by biotech companies including Anexon, Creative Biolabs and Resolvyx Inc. The majority of the larger pharmaceutical companies (e.g. Allergan, Amgen, Actelion, Bristol‐Myers Squibb) are mainly focused on developing traditional small‐molecule FPR2 modulators in a range of inflammatory disease.

### 8.2 FPR2 modulators: specialised pro‐resolving mediators in development

In the effort to develop more stable mimetics of LXA_4_, four generations of LXA_4_ analogues have been reported (as reviewed; Andrews & Godson, 2021; Fu et al., 2020). The first‐generation introduced functional groups to minimise prostaglandin dehydrogenase‐mediated degradation and prolong half‐life and bioactivity, but these compounds were susceptible to β‐oxidation (Serhan et al., 1995). The second‐generation, 3‐oxa‐LXA_4_ analogues (e.g. ZK‐192, ZK‐994) were generated with the insertion of a 3‐oxa group (Fiorucci et al., 2004). The efficacy and pharmacokinetic parameters were established in pre‐clinical models of acute topical inflammation, asthma and colitis (Gewirtz et al., 2002; Levy et al., 2007). However, the complex synthetic route hindered scalability of this approach. The third‐generation, the pyridine/benzo‐LXA_4_ analogues (O'Sullivan et al., 2007) simplified the synthetic route, with preserved anti‐inflammatory activity in a range of inflammatory models, such as hind‐limb ischaemia, peritonitis, renal fibrosis and a range of diabetic complications (kidney, liver and atherosclerosis) (Borgeson et al., 2015; Brennan, Mohan, McClelland, de Gaetano, et al., 2018; Brennan et al., 2013). Benzo‐LXA_4_ is currently being investigated as an oral rinse for the treatment of gingivitis in a phase 1 trial (trial identifier: NCT02342691) (Hasturk et al., 2021). This first‐in‐human placebo‐controlled trial demonstrated that Benzo‐LXA_4_ in a mouthwash formulation is well tolerated, reducing local inflammation and increasing circulating pro‐resolution mediators (Hasturk et al., 2021), suggesting potential applications for other inflammatory diseases. In addition, AT‐LXA_4_ and LXB_4_ have demonstrated efficacy in lowering severity of infant eczema (Wu et al., 2013), while an inhaled LXA_4_ mimetic, 5(*S*),6(*R*)‐LXA4 methyl ester and the LTA_4_ agonist, BML‐11, have both yield positive outcomes in acute and moderate episodes of asthma in children (Kong et al., 2017). Further, pro‐resolving mediators and GPCRs (including FPR2) promote resolution of inflammation in a human skin model of acute inflammation (Motwani et al., 2018). More recently, fourth‐generation LXA_4_ analogues with heteroaromatic substitutions of benzene ring (e.g. imidazole) exhibit enhanced potency (de Gaetano et al., 2019). One of the imidazole‐containing mimetics, the (*R*)‐epimer of 6C‐dimethylimidazole (AT‐01‐KG), significantly attenuated NF‐κB activity *in vitro* and displayed significant anti‐inflammatory and pre‐resolving effects in a range of inflammatory models (e.g. peritonitis, arthritis and paw swelling) *in vivo* at picomolar concentrations (Galvao et al., 2021).

ANXA1 and its fragments (e.g. annexin 1‐(2‐26), ANXA1_2‐50,_ ANXA1_2‐12_ and ANXA1_2‐6_) have been widely shown to exhibit anti‐inflammatory and pro‐resolving properties, both *in vitro* and i*n vivo*, as reviewed previously (Perretti et al., 2017; Qin et al., 2015). The N‐terminal domain of ANXA1 is critical to its biological action as well as signalling via FPR2. The most widely studied ANXA1‐derived peptide is a peptide based on the first two to 26 amino‐acids in the ANXA1 sequence, Ac‐ANXA1 (2‐26), which displays similar bioactions to the parent protein, however it does not retain an identical receptor‐specificity as ANXA1 and has much lower potency than the parent protein. Given the key cleavage site is at position 25, substitution at this site allowed the generation of metabolically stable and biologically active forms of ANXA1 and its peptide, SuperAnx‐A1 (SANXA1) and the cleavage‐resistant ANXA1_2‐50,_ CR‐ANXA1_2‐50_ (Dalli et al., 2013; Pederzoli‐Ribeil et al., 2010; Perretti et al., 2017). SANXA1 displayed stronger anti‐inflammatory effect over time compared with the parental protein (Pederzoli‐Ribeil et al., 2010). Shorter effective tripeptides (e.g. Ac‐QAW) also generated promising proof‐of‐concept data, inhibiting the growth of human colon cancer xerograph (via modulation of NF‐κB activation) and reducing postoperative neuroinflammation and cognitive changes in cardiopulmonary bypass (Zhang, Ma, et al., 2017). In addition, novel drug delivery strategies, such as nanoparticles as peptide carriers, have also been applied in efforts to improve the bioavailability and tissue distribution of ANXA1 peptides (Kamaly et al., 2013). For example, Ac‐ANXA1 (2‐26) encapsulated within targeted polymeric nanoparticles (annexin 1‐(2‐26) collagen IV nanoparticles) accelerated healing of murine colonic wounds and colitis, suggesting that development of nanoparticles containing pro‐resolving mediators may be a viable strategy for local deliveries to an injured site (Leoni et al., 2015). Similar results were shown in models of advanced atherosclerosis, in which annexin 1‐(2‐26) Collagen IV nanoparticles increased cap thickness and atherosclerotic plaques and reduced collagenase production, two key markers for atherosclerotic plaque progression (Fredman et al., 2015).

### 8.3 FPR2 modulators: small molecules in development

For the reasons discussed above, there is growing interest in the development of FPR2 small‐molecule agonists (Maciuszek et al., 2021). Compound 43, the archetypical pyrazolone FPR1/2 agonist, was discovered by Amgen during screening of compound libraries for small‐molecule FPR2 agonists, with demonstrated efficacy in a mouse ear inflammation model (Burli et al., 2006; Sogawa et al., 2011). Garcia et al. demonstrated that long‐term use of compound 43 improved cardiac function and remodelling in preclinical models of myocardial infarction, by enhancing pro‐resolution of cellular function and modulating cytokine release (Garcia et al., 2019). Some studies have however shown that administration of this small‐molecule displayed early signs of pro‐inflammation (Frohn et al., 2007; Qin et al., 2017). This may potentially be due to differences in the dosage and route of administration, or to the complex biology of FPR2 presented above. In any case, further studies are clearly warranted. Compound 17b, Z)‐N‐(3‐((4‐((3‐Chloro‐4‐fluorophenyl)amino)‐7‐methoxyquinazolin‐6‐yl)oxy)propyl)‐6‐((2‐(4‐(1‐(4‐hydroxyphenyl)‐2‐phenylbut‐1‐en‐1‐yl)phenoxy)ethyl)(methyl)amino)hexanamide, was first described in a wider series of 2‐arylacetamido‐pyridazinone FPR agonists derived from compound 43 (Cilibrizzi et al., 2009). Compound 17b was initially described as an FPR‐1‐selective agonist, based solely on intracellular Ca^2+^ mobilisation studies (Cilibrizzi et al., 2009). Subsequent *in vitro* assays have however revealed that compound 17b is an agonist at both FPR1 and FPR2 and is able to activate multiple signalling pathways (ERK1/2, cAMP and Akt, as well as intracellular Ca^2+^ mobilisation) (Qin et al., 2017). Importantly, compound 17b exhibits ~30‐fold bias away from intracellular Ca^2+^ mobilisation, at both FPR1 and FPR2, relative to other signalling endpoints and to the reference ligand (compound 43), that is, compound 17b is not as effective at stimulating the Ca^2+^ pathway when any observational or system bias is removed. The compound 17b biased‐agonist profile is associated with superior *in vitro* and *in vivo* outcomes in models of acute inflammatory insult relative to conventional agonists such as compound 43 (Qin et al., 2017), with related derivatives also evaluated *in vitro* (Deora et al., 2019).

Patents for urea derivatives serving as FPR2 modulators are largely claimed by Allergan, Inc, USA. These compounds were identified based on Ca^2+^ mobilisation assay using FLIPR^Tetra^ instruments (e.g., WO2012109544A1. 2012; WO2015042071A1. 2015). Several amide derivatives of N‐urea exhibited proof‐of‐concept efficacy in ocular inflammatory disease. There has however been an apparent shift in strategy by Allergen to develop FPR1‐selective compounds. Further, *Actelion Pharmaceuticals Ltd* developed a novel, potent small‐molecule selective FPR2 agonist, ACT‐389949 (Lind et al., 2019; Stalder et al., 2017). Results from Phase 1 studies show that ACT‐389949 is well‐tolerated in healthy subjects, but the most recent update from Adis Insights indicates ACT‐389949 has not progressed beyond Phase 1 studies, which was completed in 2013 (https://adisinsight.springer.com/drugs/800040479). Its drug‐like potential may be hampered by rapid ACT‐389949‐mediated, concentration‐dependent FPR2 receptor desensitisation and/or internalisation. Receptor internalisation was inferred from reduced cell‐surface expression of FPR2 on human monocytes and this internalisation was associated with negative pro‐inflammatory responses, including rapid (yet transient) activation of circulating cytokines, including IL‐6, IL‐8, monocyte chemoattractant protein‐1 (MCP‐1/CCR2) and TNF‐α (Lind et al., 2019; Stalder et al., 2017). More recently, *Bristol‐Myers Squibb* developed a series of piperidiones containing a 4‐phenylpyrrolidinone motif as orally active FPR2 agonists. In collaboration with Kyorin, BMS‐986235/LAR‐1219 demonstrated efficacy for prevention of heart failure in mice (Asahina et al., 2020; Garcia et al., 2021). This molecule has advanced to three Phase I clinical trials (trial identifier: NCT04301310, NCT04464577, NCT03335553).

## 9 INDICATIONS FOR FPR2‐TARGETED THERAPIES

The potential indications for FPR2‐targeted therapies that have emerged from the research literature to date are extensive and not surprisingly predominantly comprise those where an inflammatory insult (either acute or sustained) is a key component of the disorder (see Table 2). In the context of inflammatory arthritis, FPR2 activation by ANXA1 (Kao et al., 2014) or the resolvins RvD1 (Norling et al., 2016) and resolvin 3 (RvD3; Arnardottir et al., 2016) leads to clinical remission and reduces disease severity in murine arthritis, as well as affording bone (Kao et al., 2014) and cartilage (Norling et al., 2016) protection. Additionally, a recently developed FPR2 agonist designated AT‐01‐KG, a synthetic LXA_4_ mimetic, reduced markers of gouty arthritis (Kao et al., 2014), and AT‐02‐CT, a quinoxaline based LXA_4_ mimetic, promoted resolution of acute inflammation (Galvao et al., 2021).

**TABLE 2:** Selected diseases identified as amenable to FPR2‐targeted intervention *in vivo* to date

Disease/disorder	Model	Intervention	Reported impact	References
**Non‐selective FPR ligands**				
Arthritis	Acute intra‐articular carageenan (male Sprague Dawley rats)	annexin 1‐(2‐26) intra‐articular Anti‐ANXA1 mAb intra‐articular	↓ synovial lavage PMN count ↑ synovial lavage PMN count	(Yang et al., 1997)
	Adjuvant‐induced arthritis (male Sprague Dawley rats)	Intra‐articular anti‐ANXA1 mAb	↑ paw swelling and erythema	(Yang et al., 1999)
Diabetic nephropathy	High fat fed streptozotocin‐induced diabetic mice	annexin 1‐(2‐26)_26_ i.v.	↓ lipotoxicity (lipid droplet size)	(Wu et al., 2021)
Diabetic vascular complications	Streptozotocin‐induced type 1 diabetic mice	Cmpd17b i.p.	↑endothelial function, maintained vasodilatory effect under vascular injury	(Marshall et al., 2020)
Myocardial ischaemia‐induced heart failure	Coronary artery occlusion with reperfusion (male C57/Bl6 mice)	Cmpd17b i.p.	↓ infarct size, cardiac and circulating neutrophil level, cardiac apoptosis; preserved LV function	(Qin et al., 2017)
	Coronary artery permanent occlusion (male C57/Bl6 mice)	Cmpd43 oral gavage	↑ pro‐resolution cardiac M2 ↓ infarct size, ↓ LV dilatation	(Garcia et al., 2021)
	Coronary artery occlusion with reperfusion (male Sprague Dawley rats)	Cmpd43 oral gavage	↑ viable LV myocardium, ↓ LV dilatation, preserved LV function	
Stroke	Photoactivation + endotoxin‐induced microcerebrovascular thrombosis (male C57/Bl6 mice)	annexin 1‐(2‐26) i.v.	↓ thrombosis (cerebral blood flow recovery, bleeding time, platelet activation)	(Vital et al., 2020)
**FPR2‐selective ligands**				
Atherosclerosis	Streptozotocin‐induced diabetic *Apoe* ^−/−^ mice	LXA_4_, benzo‐LXA_4_ i.p.	↓ atherosclerotic plaque, vascular pro‐inflammatory gene expression	(Brennan, Mohan, McClelland, de Gaetano, et al., 2018)
	High fat fed *Apoe* ^−/−^ mice	ATL s.c.	↓ atherosclerotic plaque, macrophage infiltration into lesions; ↓ spleen and vascular cytokine/chemokine expression	(Petri et al., 2017)
Diastolic heart failure	K/BxN mouse model of arthritis	ANXA1	↑cardiac diastolic function	(Chen et al., 2021)
Liver disease	High fat fed mice	LXA_4_, benzo‐LXA_4_ i.p.	↓ liver expansion, liver function enzymes (without impact on bodyweight), ↓ liver triglycerides	(Borgeson et al., 2015)
Myocardial ischaemia	Coronary artery permanent occlusion (male C57/Bl6 mice)	ATL s.c.	↑ cardiac neutrophil clearance, ↑ cardiac pro‐resolving macrophage activation at day 5 (without impact on early cardiac dysfunction)	(Kain et al., 2017)
	Coronary artery permanent occlusion (male C57/Bl6 mice)	WRW4 s.c. (antagonist)	↑ cardiac inflammatory response; ↓ resolution mechanisms	(Kain et al., 2019)
	Coronary artery permanent occlusion (male C57/Bl6 mice)	BMS‐986235 oral gavage	↑ mouse survival and post‐MI healing, ↓left ventricular area, ↓scar area and remodelling and preserves cardiac function	(Asahina et al., 2020; Garcia et al., 2021)
	Acute ischemia/reperfusion model (male C57 Black 6 wild type and *Fpr2* ^*−/−*^ mice)	Cleavage resistant ANXA1 peptide 2‐50	Reduced infarct size and mortality	(Dalli et al., 2013)
Nephropathy	Streptozotocin‐induced diabetic *Apoe* ^−/−^ mice	LXA_4_, benzo‐LXA_4_ i.p.	↓ albuminuria, mesangial expansion and renal fibrosis, preserved renal function	(Brennan, Mohan, McClelland, Tikellis, et al., 2018)
	High fat fed mice	LXA_4_, benzo‐LXA_4_ i.p.	↓ albuminuria, mesangial expansion and renal fibrosis, preserved renal function	(Brennan, Mohan, McClelland, Tikellis, et al., 2018)
Skin inflammation	Mezerein‐induced acute ear inflammation (female NMRI mice)	ATL topical	↓ oedema, neutrophil inflammation into injury and epidermal hyperplasia	(Schottelius et al., 2002)
Stroke	Photoactivation + endotoxin‐induced microcerebrovascular thrombosis (male C57/Bl6 mice)	WRW4 s.c.	Blunted anti‐thrombotic effect of ANXA1_2‐26_	(Vital et al., 2020)
***FPR2‐modified mice***				
Atherosclerosis	High fat fed *Apoe* ^−/−^ x *Fpr2* ^−/−^ mice	Nil	*Fpr2* ^−/−^ mice exhibit ↓ atherosclerotic plaque	(Petri et al., 2017)
	Ang II‐infused Apoe^−/−^ × *Fpr2* ^−/−^ mice	Nil	*Fpr2* ^−/−^ mice exhibit ↑ aortic diameter, ↓ aortic fibrosis	(Petri et al., 2018)
Cardiac arrhythmia	*Fpr2* ^−/−^ and WT mice	Nil	*Fpr2* ^−/−^ mice exhibit ↑ cardiac expression of Na^+^, K^+^, Ca^2+^ channels and altered ECG parameters at 4 months old	(Tourki, Kain, Shaikh, et al., 2020)
Cardiomyopathy	*Fpr2* ^−/−^ and WT mice	Nil	*Fpr2* ^−/−^ mice exhibit ↑ LV chamber size, ↓ cardiac strain parameters of LV function; *Fpr2* ^−/−^ mice exhibit ↑ LV expression of macrophage trafficking, pro‐migratory chemokines, TNFα, pro‐fibrotic genes	(Tourki, Kain, Shaikh, et al., 2020) (Tourki, Kain, Pullen, et al., 2020)
Nephropathy	*Fpr2* ^−/−^ and WT mice	Nil	*Fpr2* ^−/−^ mice exhibit ↑ plasma creatinine, renal inflammation and fibrosis	(Tourki, Kain, Pullen, et al., 2020)
Obesity	*Fpr2* ^−/−^ and WT mice	Nil	*Fpr2* ^−/−^ mice exhibit ↑ food intake, fat mass, lean mass and body weight at 4 and 7 months old	(Tourki, Kain, Shaikh, et al., 2020) (Tourki, Kain, Pullen, et al., 2020)
	*Fpr2* ^−/−^ and WT mice fed high fat diet (HFD)	Nil	*Fpr2* ^−/−^ mice exhibit smaller adipocytes and ↑ lean mass but ↓ fat mass and body weight on HFD, with altered skeletal muscle thermogenic gene expression. FPR2^−/−^ mice exhibit better glycaemic control and insulin tolerance, with ↓serum +d liver triglycerides and liver lipotoxicity.	(Chen et al., 2019)

Abbreviations: ATL, aspirin‐triggered 15‐epi‐lipoxin A_4_; HFD, high fat diet; LV, left ventricle; mAb, monoclonal antibody; PMN, polymorphonuclear leukocyte; RvD, resolvin.

In recent years, cardiac pathologies have emerged as likely targets for FPR2. In human myocardium, there is evidence suggesting FPR2 may traverse from its physiological sarcolemmal location into the cytoplasm in ischaemic heart disease, based on differences in FPR2 localization in donor left ventricle (LV) samples from ischaemic versus healthy myocardium (Tourki, Kain, Pullen, et al., 2020), albeit there are also marked differences in ethnicity and concomitant medication between the two cohorts. FPR2 agonists such as ANXA1 (Tourki, Kain, Pullen, et al., 2020), Ac‐ANXA1 (2‐26) (Qin et al., 2019), RvD1 (Kain et al., 2015), BMS‐986235 (Asahina et al., 2020; Garcia et al., 2021) and LXA_4_ (Kain et al., 2017) are cardioprotective in experimental models of myocardial infarction. Additionally, the small‐molecule non‐selective FPR1/FPR2 agonist compound 17b has proven highly effective in limiting myocardial ischaemia‐reperfusion injury (Qin et al., 2017). Further evidence for an essential role for FPR2 in the heart is derived from null mice, which spontaneously develop obesity and diastolic dysfunction, as well as signs of advanced cardiac aging and reduced life‐span (Tourki, Kain, Pullen, et al., 2020). This study also demonstrated that *Fpr2* ^−/−^ mice display incomplete resolution resulting in acute decompensated heart failure post‐myocardial infarction. This was associated with lower levels of 5, 12 and 15‐lipoxygenase enzyme expression. The subsequent reduction in levels of endogenous lipid mediators in the infarcted left ventricle and spleen indicated impaired cross‐talk between the injured heart and splenic leukocytes, essential for efficient resolution of cardiac inflammation (Halade et al., 2018). It is now evident that an endogenous FPR2 agonist such as LXA_4_ can stimulate another pro‐resolving mediator (e.g., ANXA1) in a feed‐forward resolution circuit; an example of this phenomenon is evident in the context of obesity‐induced liver and kidney disease (Borgeson et al., 2015) and in the inflamed mesenteric microvasculature (Brancaleone et al., 2011). Further, 4‐week treatment of mice with recombinant ANXA1 preserves cardiac diastolic function, which is compromised in settings of inflammatory arthritis (Chen et al., 2021). Table 2 provides a summary of several key *in vivo* reports to date, but this is not intended to be an exhaustive list (with a deliberate focus on citing the earliest reports in each context where possible).

Taken together, these studies reveal promise for FPR2‐targeted therapies as a potential treatment for arthritis, cardiovascular disease and renal disease. The majority of examples provided in Table 2 are however yet to translate into clinical trial testing. Evidence for FPR2‐targeted therapies for each of these indications has been obtained from exogenous administration of peptides derived from endogenous FPR agonists, such as ANXA1 or its cleavage products, blunting inflammatory responses (and/or with a monoclonal antibody to these, in which an exacerbated inflammatory response was evident) (Vital et al., 2020; Wu et al., 2021; Yang et al., 1997, 1999), exogenous small‐molecule FPR agonists (Borgeson et al., 2015; Brennan, Mohan, McClelland, de Gaetano, et al., 2018; Kain et al., 2017; Petri et al., 2017; Qin et al., 2017; Schottelius et al., 2002) or FPR2‐deficient mice (Chen et al., 2019; Petri et al., 2017; Petri et al., 2018; Tourki, Kain, Pullen, et al., 2020; Tourki, Kain, Shaikh, et al., 2020). Lastly, given the importance of FPR2 in myeloid cell trafficking during infection and inflammation, FPRs could be used to develop novel therapeutic approaches for antibiotic development, as shown by fusion of an antibiotic‐targeting element with an FPR agonist, which enhances neutrophil clearance, providing a viable immunotherapeutic strategy to treat resistant *S. aureus* infections (Payne et al., 2021).

## 10 CONCLUSIONS AND CLOSING REMARKS

We provide here an overview of the FPR2‐subtype of formylpeptide receptors, clarifying the preferred nomenclature (while presenting insights into the sources of confusion and controversy), the complexities of intracellular signalling downstream of FPR2 activation (with particular focus on propensity for biased signalling at this particular GPCR), recent advances with respect to its structure and potential translational perspectives for this interesting pro‐resolving target.

We anticipate that the rapidly expanding translational opportunities this intriguing GPCR target offers will continue to be embraced by the wider academic research community in addition to the pharmaceutical industry and of course ourselves.

### 10.1 Nomenclature of targets and ligands

Key protein targets and ligands in this article are hyperlinked to corresponding entries in the IUPHAR/BPS Guide to PHARMACOLOGY http://www.guidetopharmacology.org and are permanently archived in the Concise Guide to PHARMACOLOGY 2021/22 (Alexander, Christopoulos, et al., 2021;Alexander, Fabbro, et al., 2021).

## CONFLICT OF INTEREST

MP has conducted commercial projects with Bristol Myers Squibb, Palatin Technologies and SynAct Pharma AS. He is on the Scientific Advisory Board of ResoTher Pharma AS, which is developing ANXA1‐derived peptides for cardiovascular settings. He consults for Bristol Myers Squibb, SynAct Pharma and TXP Pharma. CXQ consults for Shandong Hanfang Pharmaceutical Co. Ltd. All other authors have nothing to declare.

## AUTHOR CONTRIBUTIONS

CXQ, MP, and RHR conceived the review outline. CXQ, LVN, EAV, EPB, LTM, DW, MP, and RHR drafted components of the manuscript. CG, MP and RHR edited and revised the manuscript. All authors approved the final version of the manuscript.

## Data Availability

Data sharing not applicable to this article as no datasets were generated or analyzed during the current study.
